# Supplementary material for: Predicting the effectiveness of the online clinical clerkship curriculum: Development of a multivariate prediction model and validation study
Source: PLoS One. 2022 Jan 27;17(1):e0263182. doi: 10.1371/journal.pone.0263182 (PMC8794117; doi:10.1371/journal.pone.0263182)
Supplement: S1 Method — (DOCX) [file pone.0263182.s001.docx]

**Predicting the effectiveness of the online clinical clerkship curriculum: Development of a multivariate prediction model and validation study**

Naoto Kuroda, MD^*^; Anna Suzuki, MD; Kai Ozawa MD; Nobuhiro Nagai MD;

Yurika Okuyama MD; Kana Koshiishi MD; Masafumi Yamada MD;

Makoto Kikukawa, MD, MMedEd, PhD

*Corresponding author: [naoto.kuroda@wayne.edu](mailto:naoto.kuroda@wayne.edu)

**S1 Method: Detailed description of the answer options for each survey question.**

**S1 Method: Detailed description of the answer options for each survey question.**

Regarding lecture duration, there were eight answer options, arranged in 15-min increments: 1) not at all, 2) ≤15 min per lecture, 3) ≥15 min to ≤30 min per lecture, 4) ≥30 min to ≤45 min per lecture, 5) ≥45 min to ≤60 min per lecture, 6) ≥60 min to ≤75 min per lecture, 7) ≥75 min to ≤90 min per lecture, and 8) ≥90 min per lecture.

Regarding lecture frequency, there were 13 answer options: 1) not at all, 2) less than once a week, 3) about once a week, 4) about twice a week, 5) about three times a week, 6) about four times a week, 7) about five times a week, 8) about six times a week, 9) about seven times a week, 10) about eight times a week, 11) about nine times a week, 12) about ten times a week, and 13) over ten times a week.

Regarding the frequency at which students encountered technical Internet-related problems while completing an online clinical clerkship, the answers were based on a 5-point Likert scale, ranging from 1 = *not at all* to 5 = *very frequently*. The answers to the other questions were based on a 5-point Likert scale, ranging from 1 = *there were many more* *opportunities during face-to-face clinical clerkship than there were during online clinical clerkship* to 5 = *there were many more opportunities during online clinical clerkship than there were during face-to-face clinical clerkship*.
