# Supplementary material for: Predicting the effectiveness of the online clinical clerkship curriculum: Development of a multivariate prediction model and validation study
Source: PLoS One. 2022 Jan 27;17(1):e0263182. doi: 10.1371/journal.pone.0263182 (PMC8794117; doi:10.1371/journal.pone.0263182)
Supplement: S1 Table — (DOCX) [file pone.0263182.s002.docx]

**Predicting the effectiveness of the online clinical clerkship curriculum: Development of a multivariate prediction model and validation study**

Naoto Kuroda, MD^*^; Anna Suzuki, MD; Kai Ozawa MD; Nobuhiro Nagai MD;

Yurika Okuyama MD; Kana Koshiishi MD; Masafumi Yamada MD;

Makoto Kikukawa, MD, MMedEd, PhD

*Corresponding author: [naoto.kuroda@wayne.edu](mailto:naoto.kuroda@wayne.edu)

**S1 Table: Multivariate logistic regression analysis to identify the factors associated with maintaining medical students’ satisfaction with online clerkship (Level 1 in Kirkpatrick’s assessment model).**

**S1 Table: Multivariate logistic regression analysis to identify the factors associated with maintaining medical students’ satisfaction with online clerkship (Level 1 in Kirkpatrick’s assessment model).**

| Parameter | Estimate | S.E. | Pr(>\|t\|) | OR | 95% CI | |
| --- | --- | --- | --- | --- | --- | --- |
|  |  |  |  |  | L.L. | U.L. |
| Lecture duration | -0.01 | 0.03 | 0.710 | 0.99 | 0.924 | 1.055 |
| Lecture frequency | 0.05 | 0.02 | **0.010** | 1.06 | 1.013 | 1.099 |
| Quizzes | 0.29 | 0.08 | **0.001** | 1.34 | 1.134 | 1.577 |
| Assignments | 0.10 | 0.08 | 0.179 | 1.11 | 0.955 | 1.283 |
| Oral presentations | 0.17 | 0.09 | **0.046** | 1.19 | 1.003 | 1.415 |
| Observation | 0.58 | 0.17 | **0.001** | 1.79 | 1.287 | 2.499 |
| Practice | 0.59 | 0.19 | **0.002** | 1.81 | 1.247 | 2.631 |
| Interprofessional meetings | 0.42 | 0.14 | **0.003** | 1.52 | 1.150 | 2.020 |
| Interactive discussion | 0.49 | 0.09 | **<0.001** | 1.64 | 1.370 | 1.961 |
| Technical problems | -0.16 | 0.08 | **0.043** | 0.86 | 0.736 | 0.995 |
| Constant | -1.56 | 0.16 | **<0.001** | 0.21 |  |  |

S.E.: Standard error. Pr: Probability. OR: Odds ratio. CI: Confidence interval. L.L.: Lower limit. U.L.: Upper limit.

Pr < .05 indicates significance. (in **bold**)
