# Supplementary material for: Predicting the effectiveness of the online clinical clerkship curriculum: Development of a multivariate prediction model and validation study
Source: PLoS One. 2022 Jan 27;17(1):e0263182. doi: 10.1371/journal.pone.0263182 (PMC8794117; doi:10.1371/journal.pone.0263182)
Supplement: S2 Table — (DOCX) [file pone.0263182.s003.docx]

**Predicting the effectiveness of the online clinical clerkship curriculum: Development of a multivariate prediction model and validation study**

Naoto Kuroda, MD^*^; Anna Suzuki, MD; Kai Ozawa MD; Nobuhiro Nagai MD;

Yurika Okuyama MD; Kana Koshiishi MD; Masafumi Yamada MD;

Makoto Kikukawa, MD, MMedEd, PhD

*Corresponding author: [naoto.kuroda@wayne.edu](mailto:naoto.kuroda@wayne.edu)

**S2 Table: Multivariate logistic regression analysis to identify the factors associated with maintaining medical students’ motivation during online clerkship (Level 2a in Kirkpatrick’s assessment model).**

**S2 Table: Multivariate logistic regression analysis to identify the factors associated with maintaining medical students’ motivation during online clerkship (Level 2a in Kirkpatrick’s assessment model).**

| Parameter | Estimate | S.E. | Pr(>\|t\|) | OR | 95% CI | |
| --- | --- | --- | --- | --- | --- | --- |
|  |  |  |  |  | L.L. | U.L. |
| Lecture duration | 0.01 | 0.03 | 0.877 | 1.01 | 0.942 | 1.072 |
| Lecture frequency | 0.03 | 0.02 | 0.124 | 1.03 | 0.991 | 1.075 |
| Quizzes | 0.26 | 0.08 | **0.002** | 1.30 | 1.106 | 1.532 |
| Assignments | -0.03 | 0.07 | 0.725 | 0.97 | 0.843 | 1.126 |
| Oral presentations | 0.31 | 0.09 | **<0.001** | 1.37 | 1.155 | 1.621 |
| Observation | 0.43 | 0.17 | **0.010** | 1.53 | 1.107 | 2.122 |
| Practice | 0.66 | 0.19 | **0.001** | 1.93 | 1.328 | 2.804 |
| Interprofessional meetings | 0.50 | 0.14 | **0.001** | 1.64 | 1.240 | 2.178 |
| Interactive discussion | 0.34 | 0.09 | **<0.001** | 1.40 | 1.173 | 1.676 |
| Technical problems | -0.27 | 0.08 | **<0.001** | 0.76 | 0.658 | 0.887 |
| Constant | -1.19 | 0.15 | **<0.001** | 0.30 |  |  |

S.E.: Standard error. Pr: Probability. OR: Odds ratio. CI: Confidence interval L.L.: Lower limit. U.L.: Upper limit.

Pr < .05 indicates significance. (in **bold**)
