# Supplementary material for: Predicting the effectiveness of the online clinical clerkship curriculum: Development of a multivariate prediction model and validation study
Source: PLoS One. 2022 Jan 27;17(1):e0263182. doi: 10.1371/journal.pone.0263182 (PMC8794117; doi:10.1371/journal.pone.0263182)
Supplement: S3 Table — (DOCX) [file pone.0263182.s004.docx]

**Predicting the effectiveness of the online clinical clerkship curriculum: Development of a multivariate prediction model and validation study**

Naoto Kuroda, MD^*^; Anna Suzuki, MD; Kai Ozawa MD; Nobuhiro Nagai MD;

Yurika Okuyama MD; Kana Koshiishi MD; Masafumi Yamada MD;

Makoto Kikukawa, MD, MMedEd, PhD

*Corresponding author: [naoto.kuroda@wayne.edu](mailto:naoto.kuroda@wayne.edu)

**S3 Table: The final model using multivariate logistic regression analysis to predict medical students’ satisfaction with online clerkship (Level 1 in Kirkpatrick’s assessment model).**

**S3 Table: The final model using multivariate logistic regression analysis to predict medical students’ satisfaction with online clerkship (Level 1 in Kirkpatrick’s assessment model).**

| Parameter | Estimate | S.E. | Pr(>\|t\|) | OR | 95% CI | |
| --- | --- | --- | --- | --- | --- | --- |
|  |  |  |  |  | L.L. | U.L. |
| Lecture frequency | 0.05 | 0.02 | **0.007** | 1.05 | 1.013 | 1.088 |
| Quizzes | 0.32 | 0.08 | **<0.001** | 1.38 | 1.178 | 1.615 |
| Oral presentations | 0.19 | 0.09 | **0.027** | 1.21 | 1.023 | 1.437 |
| Observation | 0.58 | 0.17 | **0.001** | 1.79 | 1.289 | 2.491 |
| Practice | 0.59 | 0.19 | **0.002** | 1.80 | 1.238 | 2.606 |
| Interprofessional meetings | 0.42 | 0.14 | **0.004** | 1.52 | 1.144 | 2.008 |
| Interactive discussion | 0.49 | 0.09 | **<0.001** | 1.64 | 1.368 | 1.957 |
| Technical problems | -0.16 | 0.08 | **0.038** | 0.85 | 0.735 | 0.991 |
| Constant | -1.49 | 0.13 | **<0.001** | 0.23 |  |  |

S.E.: Standard error. Pr: Probability. OR: Odds ratio. CI: Confidence interval L.L.: Lower limit. U.L.: Upper limit.

Pr < .05 indicates significance. (in **bold**)
