# Supplementary material for: Predicting the effectiveness of the online clinical clerkship curriculum: Development of a multivariate prediction model and validation study
Source: PLoS One. 2022 Jan 27;17(1):e0263182. doi: 10.1371/journal.pone.0263182 (PMC8794117; doi:10.1371/journal.pone.0263182)
Supplement: S4 Table — (DOCX) [file pone.0263182.s005.docx]

**Predicting the effectiveness of the online clinical clerkship curriculum: Development of a multivariate prediction model and validation study**

Naoto Kuroda, MD^*^; Anna Suzuki, MD; Kai Ozawa MD; Nobuhiro Nagai MD;

Yurika Okuyama MD; Kana Koshiishi MD; Masafumi Yamada MD;

Makoto Kikukawa, MD, MMedEd, PhD

*Corresponding author: [naoto.kuroda@wayne.edu](mailto:naoto.kuroda@wayne.edu)

**S4 Table: The final model using multivariate logistic regression analysis to predict medical students’ motivation during online clerkship (Level 2a in Kirkpatrick’s assessment model).**

**S4 Table: The final model using multivariate logistic regression analysis to predict medical students’ motivation during online clerkship (Level 2a in Kirkpatrick’s assessment model).**

| Parameter | Estimate | S.E. | Pr(>\|t\|) | OR | 95% CI | |
| --- | --- | --- | --- | --- | --- | --- |
|  |  |  |  |  | L.L. | U.L. |
| Quizzes | 0.27 | 0.08 | **0.001** | 1.31 | 1.122 | 1.529 |
| Oral presentations | 0.33 | 0.08 | **<0.001** | 1.39 | 1.180 | 1.643 |
| Observation | 0.42 | 0.16 | **0.010** | 1.52 | 1.104 | 2.105 |
| Practice | 0.65 | 0.19 | **0.001** | 1.92 | 1.320 | 2.786 |
| Interprofessional meetings | 0.50 | 0.14 | **0.001** | 1.64 | 1.241 | 2.179 |
| Interactive discussion | 0.36 | 0.09 | **<0.001** | 1.43 | 1.198 | 1.707 |
| Technical problems | -0.25 | 0.07 | **0.001** | 0.78 | 0.674 | 0.903 |
| Constant | -1.11 | 0.11 | **<0.001** | 0.33 |  |  |

S.E.: Standard error. Pr: Probability. OR: Odds ratio. CI: Confidence interval L.L.: Lower limit. U.L.: Upper limit.

Pr < .05 indicates significance. (in **bold**)
