# Supplementary material for: Predicting the effectiveness of the online clinical clerkship curriculum: Development of a multivariate prediction model and validation study
Source: PLoS One. 2022 Jan 27;17(1):e0263182. doi: 10.1371/journal.pone.0263182 (PMC8794117; doi:10.1371/journal.pone.0263182)
Supplement: Attachment — Submitted filename: rebuttal_letter_nk5.docx [file pone.0263182.s008.docx]

Journal Requirements:

When submitting your revision, we need you to address these additional requirements.

1. Please ensure that your manuscript meets PLOS ONE's style requirements, including those for file naming. The PLOS ONE style templates can be found at 
https://journals.plos.org/plosone/s/file?id=wjVg/PLOSOne_formatting_sample_main_body.pdf and 
https://journals.plos.org/plosone/s/file?id=ba62/PLOSOne_formatting_sample_title_authors_affiliations.pdf

**→We confirmed it.**

2. In your Data Availability statement, you have not specified where the minimal data set underlying the results described in your manuscript can be found. PLOS defines a study's minimal data set as the underlying data used to reach the conclusions drawn in the manuscript and any additional data required to replicate the reported study findings in their entirety. All PLOS journals require that the minimal data set be made fully available. For more information about our data policy, please see <http://journals.plos.org/plosone/s/data-availability>.

**→We attached the data set of this study as supplementary files.**

Upon re-submitting your revised manuscript, please upload your study’s minimal underlying data set as either Supporting Information files or to a stable, public repository and include the relevant URLs, DOIs, or accession numbers within your revised cover letter. For a list of acceptable repositories, please see http://journals.plos.org/plosone/s/data-availability#loc-recommended-repositories. Any potentially identifying patient information must be fully anonymized.

Important: If there are ethical or legal restrictions to sharing your data publicly, please explain these restrictions in detail. Please see our guidelines for more information on what we consider unacceptable restrictions to publicly sharing data: http://journals.plos.org/plosone/s/data-availability#loc-unacceptable-data-access-restrictions. Note that it is not acceptable for the authors to be the sole named individuals responsible for ensuring data access.

We will update your Data Availability statement to reflect the information you provide in your cover letter.

Additional Editor Comments:

The concept of developing a predictive model for evaluating effectiveness of the online clinical clerkship curriculum is important, interesting and very relevant.
You will need to add more details on the questionnaire validation process.

**→Thank you for your comments. We added the following details on the questionnaire validation process:**

**We developed the survey questions with a focus on content validity through an iterative process within the team, which included a medical educator. In addition, we administered the survey to a pilot group of 30 medical students. During a semi-structured interview after the survey was administered, these students were asked to provide feedback on the clarity of formulation of the items and on the format of the questionnaire.**

A brief summary of the curricular model followed (especially for year 5 and 6 )normally needs to be added to put things in context
**→As your suggestion, we added the information about Japanese medical education as followings:**

**The Japanese undergraduate medical education curriculum is six years period. It usually consists of four/three and half years of preclinical education and two/two and half years of clinical clerkship. Clinical clerkship has medical students participate as members of a medical team in a variety of clinical settings. Medical students can acquire clinical competencies through clinical clerkship.**

Limitations need to be elaborated, would have been especially good if faculty perspective was also incorporated

**→As your comments, we added discussion about the factors associated with faculties in the limitation part as followings:**

**The third limitation is that qualitative factors on the part of the faculty were not considered in the current model because the current study was a quantitative survey. For example, the content and quality of lectures, the faculty's skills of online facilitation might also affect the students' satisfaction and motivation.**

Finally, the discussion needs to elaborate on how far the results can be extrapolated to other regions

**→As your suggestion, we added the discussion how far the results can be extrapolated to other regions as followings:**

**We believe that our prediction model can be applied in other countries’ online clinical clerkship curriculum or post-COVID-19 pandemic. However, we need to evaluate our model’s validity in other countries and post-COVID-19 pandemic in future studies.**

Reviewers' comments:

**Comments to the Author**

Review Comments to the Author

Reviewer #1: Article is recommended for submission since it meets all the criteria of the journal. The tool for validation of online clerkship is something that is a requisite today. The statistical works and the language of the paper are acceptable. The reference styling is acceptable.

**→Thank you for your favorable comments.**

Reviewer #2: The study is very relevant considering the pandemic and could be very useful to Medical Colleges all over the world to evaluate and improve on their curricula.
A potential source of the results not reflecting actual improvement in the curricula, is the fact, that in the current scenario online learning is more of a necessity rather than an option. The student satisfaction being high could be reflecting their greater comfort rather than better learning outcomes as the study is only assessing student satisfaction and has no information regarding correlation with exam scores or patient outcomes. More research might be required in these avenues before using this study as a tool to modify curricula.

**→Thank you for your comments. As you pointed out, the clear limitation of this study is that we did not investigate the correlation with exam scores or patient’s outcomes. We added this limitation as follows:**

**Therefore, more research might be required to show the correlation with exam scores or patient outcomes before using this study as a tool to modify curricula.**
